# An extract from whole *Coffea arabica* coffee cherry improves time trial performance, but not muscle glycogen resynthesis, in trained cyclists

**DOI:** 10.1080/15502783.2026.2682323

**Published:** 2026-06-03

**Authors:** George F. Pavis, Ronald L. Kingma, Boris V. Nemzer, Nebiyu Abshiru, Benjamin T. Wall, Francis B. Stephens

**Affiliations:** a Department of Public Health and Sport Sciences, Nutritional Physiology Research Group, Public Health & Sports Sciences, University of Exeter, Exeter, Devon, UK; b Research and Development, VDF FutureCeuticals Inc., Momence, IL, USA; c Department of Food Science and Human Nutrition, University of Illinois at Urbana-Champaign, Urbana, IL, USA

**Keywords:** Caffeine, muscle glycogen, recovery, endurance performance

## Abstract

**Background:**

This study aimed to test the hypothesis that a whole *Coffea arabica* coffee cherry extract concentrated in caffeine and polyphenols would increase exercise performance and muscle glycogen resynthesis.

**Methods:**

Twelve trained cyclists (11 male, 1 female; V̇O_2max_: 55 ± 2 mL·kg^−1^·min^−1^) took part in a double blind, randomized, placebo-controlled, cross-over trial. Participants consumed either a coffee cherry extract supplement containing 200 mg caffeine and 15 mg polyphenols (CB) or placebo (PLA), before exercising for 30 min on a cycle ergometer at 79 ± 1% V̇O_2max_ followed by a 15 min time trial (TT1). A second supplement was then consumed with 1 g·kg bm^−1^ carbohydrate and a second time trial (TT2) was performed the following morning. Blood samples were collected throughout, and muscle biopsies were collected immediately after TT1 (0 h), and at 4 and 24 h.

**Results:**

Plasma caffeine and chlorogenic acid concentrations increased during exercise and subsequent recovery in CB compared to PLA (*p* < 0.001). Total work performed during TT1 was 4.6% ± 1.5% greater with CB than PLA (3.14 ± 0.15 vs. 3.02 ± 0.17 kJ·kg^−1^; Hedge's g [95% confidence interval] = 0.8 [0.2, 1.4]; *p* < 0.05). Perceived exertion was lower during steady-state exercise with CB (*p* < 0.05); respiratory exchange ratio did not differ. From 0 to 4 h recovery, muscle glycogen content increased similarly in PLA and CB by 64 ± 13 and 44 ± 10 mmol·kg dw^−1^, respectively, and by 53 ± 8 and 45 ± 10 mmol·kg dw^−1^ from 4 to 24 h (*P* < 0.001).

**Conclusions:**

We report that an extract from whole *Coffea arabica* cherry containing a low dose of caffeine and polyphenols is ergogenic but was not shown to enhance muscle glycogen resynthesis following post-exercise consumption of 1 g·kg bm^−1^ carbohydrate.

**Clinical trials registration:** Registered with clinicaltrials.gov on 10/01/2022 as NCT05404841 (URL: https://clinicaltrials.gov/study/NCT05404841).

## 1. Background

Caffeine-containing supplements are among the most commonly used nutritional supplements by recreational and elite athletes, optimizing exercise performance and/or reducing effort perception [1–3]. Coffee beans are the main source of commercially available caffeine, but they also contain a diverse array of other phytonutrients that may be beneficial for exercise performance and recovery [4,5]. While historically the coffee fruit has been discarded and is not commonly utilized in nutritional supplements, the whole coffee cherry from which the bean is derived is also rich in polyphenols. For example, in-depth profiling of *Coffea arabica* coffee cherries (including both the coffee bean and the cherry husk) identified up to 219 compounds, consisting of isomers of chlorogenic acids, flavonoids, alkaloids, eicosanoyl-5-hydroxytryptamide, atractyligenin, and carboxyatractyligenin derivatives [6]. Despite this, there is a paucity of data addressing whether whole coffee cherry extracts can have an ergogenic effect.

High doses (>6 mg·kg body mass (bm)^−1^) of caffeine improve exercise performance and influence substrate metabolism [1,7,8]. Moreover, post-exercise ingestion of 8 mg·kg bm^−1^ caffeine, either alone or in a coffee drink, with 4 g⋅kg bm^−1^ of carbohydrate markedly increases muscle glycogen resynthesis by more than 60% over 4 h [9,10]. However, high doses of caffeine can have detrimental side effects [7] and do not appear to be any more ergogenic than lower doses (1.5 to 3 mg⋅kg bm^−1^) [1,7,8]. Given that coffee cherries include caffeine naturally complexed with polyphenols [11], and that polyphenols can support a broad array of physiological modulations [12], coffee cherry extracts may be an effective intervention to provide a low dose of caffeine for exercise performance and recovery.

Here, we aimed to test the hypothesis that ingesting a whole *Coffea arabica* coffee cherry extract before 30 min of high-intensity steady-state (SS) exercise in trained cyclists would reduce effort perception and increase performance in a subsequent 15-min time trial. In addition, we hypothesized that an additional dose consumed immediately after exercise would enhance muscle glycogen resynthesis and time trial performance the following day.

## 2. Methods

### 2.1. Participants

Twelve trained cyclists (11 male, 1 female; age: 29 ± 2 years; height: 179 ± 2 cm; body mass: 76 ± 3 kg; V̇O_2max_: 55 ± 2 mL·kg bm^−1^·min^−1^; W_max_: 4.48 ± 0.19 W·kg^−1^) with competitive time trial and/or triathlon experience and self-reported habitual caffeine use took part in this study. All participants met the classification of being Tier 2 athletes [13], and habitual caffeine use was chosen to minimize the risk of adverse events from unaccustomed caffeine intake. The sample size was determined *a priori* and informed by our previous work [14]; assuming an effect size of the current intervention on time trial performance (our primary outcome) of d_z_ = 0.9, *α* = 0.05, and power of 80%; 12 participants were deemed necessary. To account for potential dropouts, we sought to recruit 14 individuals. Exclusion criteria were: diagnosed metabolic impairment; cardiovascular disease; chronic use of prescribed or over-the-counter pharmaceuticals; pregnant; musculoskeletal injury within 6 months prior to enrollment; allergy to lidocaine; habitual smoker. Written consent was obtained from all participants following full explanation of the experimental procedures, which were approved by the University of Exeter's Sport and Health Sciences Ethics Committee (reference no. 2021-M-38). This study was registered at ClinicalTrials.gov as NCT05404841.

### 2.2. Study design

**Figure 1.**
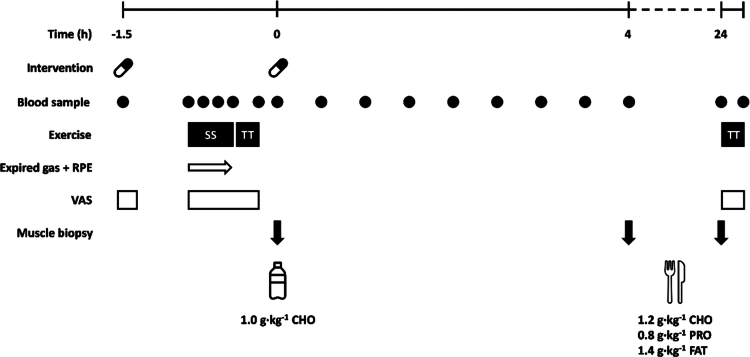
Graphical representation of the experimental trial. Time (h) shown relative to the start of the recovery period. SS, steady state exercise; TT, time trial; CHO, carbohydrate; PRO, protein.

Participants attended the Nutritional Physiology Research Unit at the University of Exeter for two pre-experimental and two experimental trials. The pre-experimental trials were to determine maximal oxygen consumption (V̇O_2max_) and to familiarize participants with the exercise testing protocol. The experimental trials (Figure 1) were conducted in randomized, counterbalanced order using a double-blinded, cross-over design. Participants were provided with either an encapsulated oral supplement (CB) containing 200 mg caffeine and ∼15 mg polyphenols from whole *Coffea arabica* coffee cherry extract (including both the coffee bean and cherry husk; FutureCeuticals, IL, USA), or an identical micro-crystalline cellulose placebo (PLA). All trials were each separated by ≥7 days (mean ± SD: 16 ± 12; range 7 to 49 days) and conducted in the overnight fasted state to minimize any influence of prior feeding on metabolism. In the 24 h prior to each experimental trial, participants were encouraged to consume a low-carbohydrate diet and abstain from caffeine and strenuous exercise. Diet and physical activity were recorded in the 24 h prior to the familiarization and were repeated as closely as possible before each experimental trial.

### 2.3. Pre-experimental trials

Upon successful screening, anthropometric measures were obtained, and participants performed a step incremental cycling test on an electronically braked cycle ergometer (Lode Excalibur Sport, Groningen, The Netherlands) as per our prior work [14]. The test started at either 100 or 150 W depending on participants' self-reported cycling ability, increasing in 40 W increments every 3 min until volitional exhaustion. Pulmonary gas exchange was measured breath-by-breath (Metalyzer 3B, Cortex Biophysik GmbH, Leipzig, Germany) and subsequently averaged into 5 s periods. We defined V̇O_2max_ as the highest 15 s mean value attained before fatigue. After a 15 min rest, a confirmation test began whereby participants cycled at 100 W for 3 min, then continued the step incremental protocol from the stage at which they fatigued. Achievement of V̇O_2max_ was accepted when a plateau in oxygen consumption occurred in the initial trial, and/or was not surpassed in the confirmation test. Maximum work capacity (W_max_) was calculated from the first incremental test as W_max_ (W) = W′ + (40 · *t*/180), where W′ is the highest workload completed, and t (s) is the time attained in the final, incomplete, stage at exhaustion. All equipment settings and positions were recorded and repeated for subsequent visits.

The familiarization trial consisted of a 30 min steady state (SS) cycle followed by a 15 min time trial (TT). During the SS cycle, workload was clamped irrespective of cadence to maintain a constant power output. V̇O_2_ was measured continuously to allow workload adjustments to elicit 80% V̇O_2max_. Following a 5 min rest after the SS, participants performed a 15 min TT under the instruction of completing as much work as possible. Participants were blinded to their performance with no encouragement, but were alerted when they had completed 5 and 10 min of the TT. During the TT, power output was proportional to cadence, whereby resistance was applied to take into account each individual's preferred cadence and power output during the SS [15].

### 2.4. Experimental trials

Participants reported to the laboratory at 0800 h. Urine osmolality was determined in a baseline mid-stream urine sample (Osmomat 030-D, Gonotec, Berlin, Germany), followed by a body mass measurement. A baseline 10 mL blood sample was collected from a cannula placed into an antecubital vein, before participants consumed their allocated supplement. This was administered in capsule form and consumed with 50 mL water. After a 40 min rest, a second blood sample was obtained, and participants began a warm-up of cycling at 100 W for 5 min. The 30 min SS cycle began immediately after. Heart rate (HR) and expired gases were obtained throughout the SS cycle. After a 5 min rest, participants completed a 15 min TT as described above. Additional blood samples were collected at 10, 20, and 30 min of the SS and immediately post-TT, alongside rating of perceived exertion (RPE) and measurements of motivation, alertness, and gastrointestinal (GI) discomfort. Borg's 15-point rating of perceived exertion [16] was used to determine RPE, and participants indicated motivation, alertness, and GI discomfort using separate 10 cm visual analog scales (VAS) for each parameter. Participants were fan-cooled whilst cycling.

Following exercise, a second mid-stream urine sample and body mass measurement were obtained. Participants then lay supine while a percutaneous biopsy of the *m. vastus lateralis* was obtained under local anesthesia (2% lidocaine). An additional dose of the allocated supplement was provided alongside 1 g·kg bm^−1^ dextrose monohydrate beverage (Myvegan, Manchester, UK) as an 18% solution in water, marking the start of the recovery period (0 h). Additional venous blood samples were obtained every 30 min, thereafter, with a second muscle biopsy taken after 4 h of recovery. Participants then left the laboratory with a controlled diet for the remainder of the day. All food was weighed out and individually packaged, providing 1.2 g·kg bm^−1^ carbohydrate, 0.8 g·kg bm^−1^ protein, and 1.4 g·kg bm^−1^ fat. Participants were informed of the importance of diet adherence and returned a checklist indicating whether each item was consumed. No other foods or energy-containing beverages were permitted.

Participants returned to the laboratory the following morning in the overnight fasted state and a final mid-stream urine sample was collected. A pre-exercise blood sample was collected via a cannula in the antecubital vein, followed by a third muscle biopsy at exactly 24 h after consuming the dextrose solution. Participants then completed a 5 min warm-up at 100 W on the cycle ergometer, before completing a second TT in a similar fashion as the previous day. A final venous blood sample was then obtained.

### 2.5. Sample collection

From a 10 mL collection of whole blood, one aliquot (20 μL) was suspended in sodium-heparinized capillary tubes and placed in glucose/lactate solution cups to determine blood glucose and lactate concentration (Biosen C-line; EKF Diagnostics, Cardiff, UK). The remaining blood was aliquoted into vacutainers containing lithium heparin, and serum separator gel (BD Company, NJ, USA). Plasma samples were processed immediately, while serum stood at room temperature for 30 min, before centrifugation at 2950 × *g* for 10 min at 4 °C. Plasma and serum were aliquoted and stored at −80 °C for further analysis.

Muscle biopsies were obtained under local anesthesia (2% lidocaine) from the midsection of the m. *vastus lateralis* by the Bergström needle technique modified for suction [17]. Repeated samples within the same trial were obtained from the same leg, ~2.5 cm proximal to the previous site. The leg to be biopsied was pre-determined according to the randomization plan, which was counterbalanced for leg dominance. All samples were rapidly dissected of visible fat and connective tissue, frozen in liquid nitrogen, and stored at −80 °C until subsequent analysis.

### 2.6. Plasma and serum analyses

Plasma ketone and serum non-esterified fatty acid (NEFA) concentrations were quantified using colorimetric assays according to manufacturers' instructions (β-Hydroxybutyrate Assay Kit, Sigma-Aldrich, MO, USA; NEFA assay FA115, Randox, Crumlin, UK).

Plasma caffeine and paraxanthine concentrations were quantified via high-performance liquid chromatography (HPLC) [18]. Briefly, 250 μL plasma was deproteinized on ice with 250 μL perchloric acid (0.8 M) before centrifugation. The supernatant was then adjusted to pH 5.0 with 4  M sodium hydroxide and injected into a Flexar LC system (Perkin Elmer, MA, USA). Known standards and deproteinized samples were eluted isocratically through a reverse-phase C18HD analytical column (Perkin Elmer), with peaks detected by ultraviolet absorbance at 274 nm. All samples for each participant were run in a single batch to decrease between-run variability.

Plasma chlorogenic acids were analyzed with a Q-Exactive Hybrid Quadrupole-Orbitrap mass spectrometer (Thermo Scientific) coupled with a Vanquish Neo UHPLC system (Thermo Scientific) as described previously [6]. Briefly, 300 μL plasma was spiked with 20 μL internal standard and vortexed with 1500 μL methanol to precipitate proteins. Following centrifugation, the supernatant was dried under N_2_ gas and reconstituted in 100 μL of MS-grade water with 0.1% formic acid before being injected into the system as two replicates in negative mode.

### 2.7. Muscle analyses

Muscle glycogen content was determined in ~30 mg wet weight muscle tissue. Samples were freeze-dried for 24 h and powdered by hand, with visible blood and connective tissue removed. Muscle powder (2.1 ± 0.1 mg) was hydrolyzed in 500 μL 1 M hydrochloric acid at 100 °C for 3 h and glycosyl units quantified immediately using an automated glucose analyzer (YSI 2900 Biochemistry Analyzer; Yellow Springs Instruments, Yellow Springs, OH).

### 2.8. Statistics

A paired samples *t*-test was used to test for differences in TT performance. All other data (blood and muscle metabolites, muscle glycogen, motivation, alertness, GI discomfort, HR, RPE, respiratory exchange ratio (RER), urine osmolality, and body mass) were analyzed by repeated-measures two-way analysis of variance (ANOVA)s. GI discomfort presented considerable skew toward zero, and so the statistical analysis was performed on log-transformed data to ensure normal distribution. The two-way ANOVAs performed on blood lactate and glucose, and plasma metabolites were run separately for baseline to end of SS, end of SS to end of TT, and recovery periods. Tukey corrections for multiple comparisons were applied to identify post hoc differences. Statistical analysis was performed using GraphPad Prism 9 (GraphPad Software, Inc., San Diego, CA, USA). All data are presented as mean ± SEM to represent the precision of the condition means, which is of primary interest in this mechanistic context, with *p* < 0.05 indicating statistical significance. For TT1 performance (primary outcome), magnitude of the change is also presented as effect size (Hedge's *g* [with 95% confidence intervals]) [19].

## 3. Results

### 3.1. Descriptive results

Each dose of CB provided 200 mg caffeine (2.7 ± 0.1 mg·kg bm^−1^). Body mass decreased from baseline to after the first TT, from 76.4 ± 2.7 to 75.4 ± 2.7 kg with PLA and from 76.2 ± 2.8 to 75.1 ± 2.7 kg with CB (time *p* < 0.001). However, this change was unaffected by CB (supplement and interaction *p* > 0.05). Urine osmolality was unchanged from baseline to after the first TT with both PLA and CB, but was greater with CB than PLA prior to the second TT after 24 h recovery (0.615 ± 0.093 vs. 0.336 ± 0.065 Osmol·kg^−1^; interaction *p* < 0.01). Laboratory temperature (19.7 °C ± 0.3 °C) and humidity (53% ± 3%) did not differ between PLA or CB trials. Given that muscle glycogen resynthesis was a secondary hypothesis, some participants elected not to have a muscle biopsy taken. Muscle biopsy samples, therefore, were collected from *n* = 8 participants. In the PLA trial, three participants correctly identified their supplement allocation. In the CB trial, four participants correctly identified the allocation.

### 3.2. Plasma caffeine and paraxanthine concentrations

**Figure 2.**
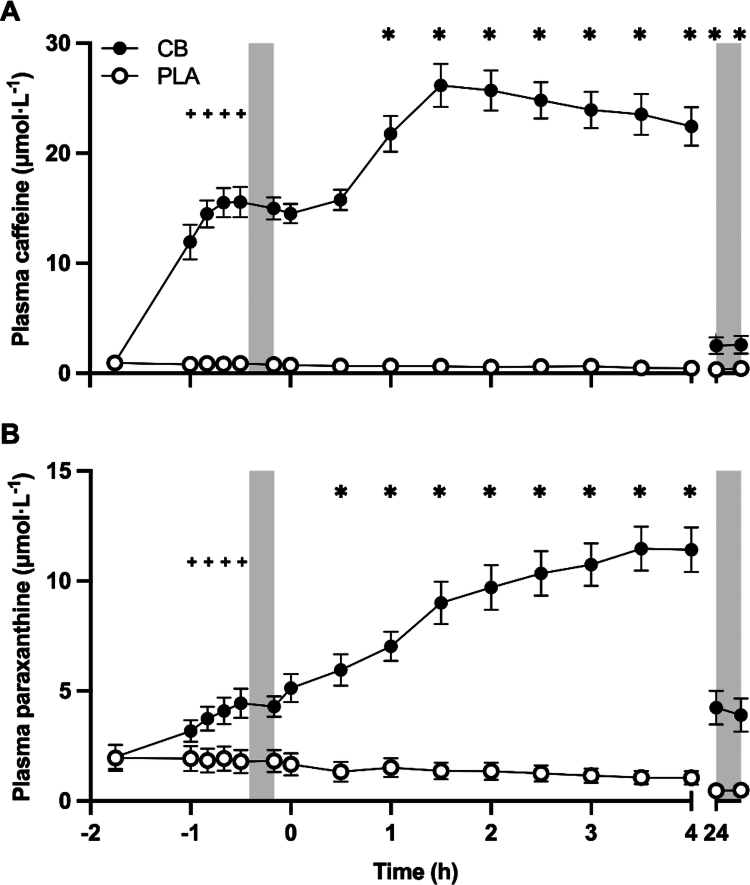
Plasma concentrations of (A) caffeine, and (B) paraxanthine before and after consuming a multi-ingredient supplement containing 200 mg caffeine (CB; filled symbols) or placebo (PLA; open symbols) in trained cyclists (*n* = 12). Data analyzed by two-way ANOVAs, run separately for baseline to end of steady state, end of steady state to end of time trial, and from 0 to 24 h of recovery, but plotted together for presentation purposes. Post hoc differences within interaction effects denoted by ^+^ *p* < 0.001 significantly different to baseline during steady state in CB; *p < 0.01 significantly different to start of recovery (0 h) in CB.

There were no differences in plasma caffeine concentrations between PLA and CB in the baseline blood sample (Figure 2A). From baseline, plasma caffeine concentration remained unchanged with PLA throughout SS, but was elevated by CB immediately prior to, and at 10, 20 and 30 mins of SS (interaction *p* < 0.001; all timepoints increased from baseline, and greater than PLA; post hoc *p* < 0.001). Before and after the first TT, plasma caffeine concentrations were significantly greater with CB than with PLA (supplement *p* < 0.001), irrespective of time (both time and interaction *p* > 0.05). During recovery, plasma caffeine concentrations remained unchanged with PLA. However, plasma caffeine further increased from 0 to 4 h of recovery with CB (interaction *p* < 0.001; all timepoints within 4 h increased, and greater than PLA; post hoc *p* < 0.01). The following day, plasma caffeine concentrations decreased from 0 h of recovery (post hoc *p* < 0.001), and were no longer different to PLA (post hoc *p* > 0.05). Plasma caffeine concentrations did not differ before versus after the second TT (post hoc *p* > 0.05).

There were no differences in plasma paraxanthine concentrations between PLA and CB in the baseline blood sample (Figure 2B). From baseline, plasma paraxanthine concentrations remained unchanged with PLA throughout SS, but increased with CB immediately prior to, and at 10, 20, and 30 mins of SS (interaction *p* < 0.001; all timepoints increased from baseline, and greater than PLA; post hoc *p* < 0.001). Before and after the first TT, plasma paraxanthine concentrations were significantly greater with CB than with PLA (supplement *p* < 0.001), irrespective of time (both time and interaction *p* > 0.05). During recovery, plasma paraxanthine concentrations remained unchanged with PLA. However, plasma paraxanthine further increased from 0 to 4 h of recovery with CB (interaction *p* < 0.001; all timepoints within 4 h increased, and greater than PLA; post hoc *p* < 0.01). The following day, plasma paraxanthine concentrations did not differ versus 0 h of recovery (post hoc *p* > 0.05) but remained greater than PLA (post hoc *p* < 0.05). Plasma paraxanthine concentrations remained unchanged before versus after the second TT (post hoc *p* > 0.05).

**Figure 3.**
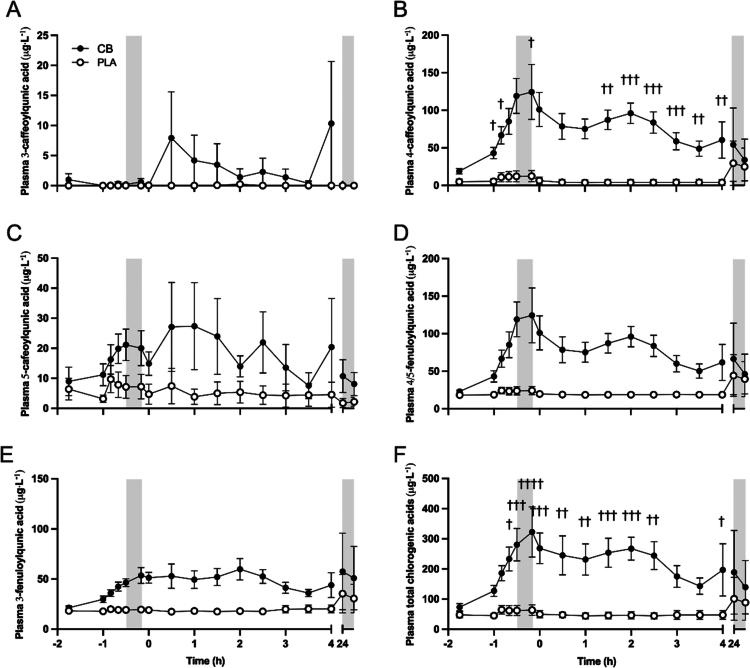
Plasma concentrations of (A) 3-caffeoylquinic acid, (B) 4-caffeoylquinic acid, (C) 5-caffeoylquinic acid, (D) 4/5-fenuloylquinic acid, (E) 3-fenuloylquinic acid, and (F) total chlorogenic acids, before and after consuming a multi-ingredient supplement containing 200 mg caffeine (CB; filled symbols) or placebo (PLA; open symbols) in trained cyclists (*n* = 12). Data analyzed by two-way ANOVAs, run separately for baseline to end of steady state, end of steady state to end of time trial, and from 0 to 24 h of recovery, but plotted together for presentation purposes. ^†^ *p* < 0.05, ^††^ *p* < 0.01, ^†††^ *p* < 0.001, and ^††††^ *p* < 0.0001 CB significantly different to PLA.

Plasma concentrations of 4-caffeoylquinic acid (*p* < 0.0001; Figure 3B), 5-caffeoylquinic acid (*p* < 0.05; Figure 3C), 4/5-feruloylquinic acid (*p* < 0.001; Figure 3D), 3-feruloylquinic acid (*p* < 0.0001; Figure 3E), and total chlorogenic acids (*p* < 0.0001; Figure 3F) were greater throughout SS, TT, and recovery in CB compared to PLA, whereas plasma 3-caffeoylquinic acid concentration (Figure 3A) was not different between CB and PLA at any timepoint. Plasma 4-caffeoylquinic acid (interaction *p* < 0.05) and total chlorogenic acids (interaction *p* < 0.01), in particular, increased above PLA by up to 10-fold at a peak 1.5 h after ingestion of each of the CB drinks. There were no differences between CB and PLA in any of the chlorogenic acids before or after the TT the following day.

### 3.3. Steady state exercise

**Figure 4.**
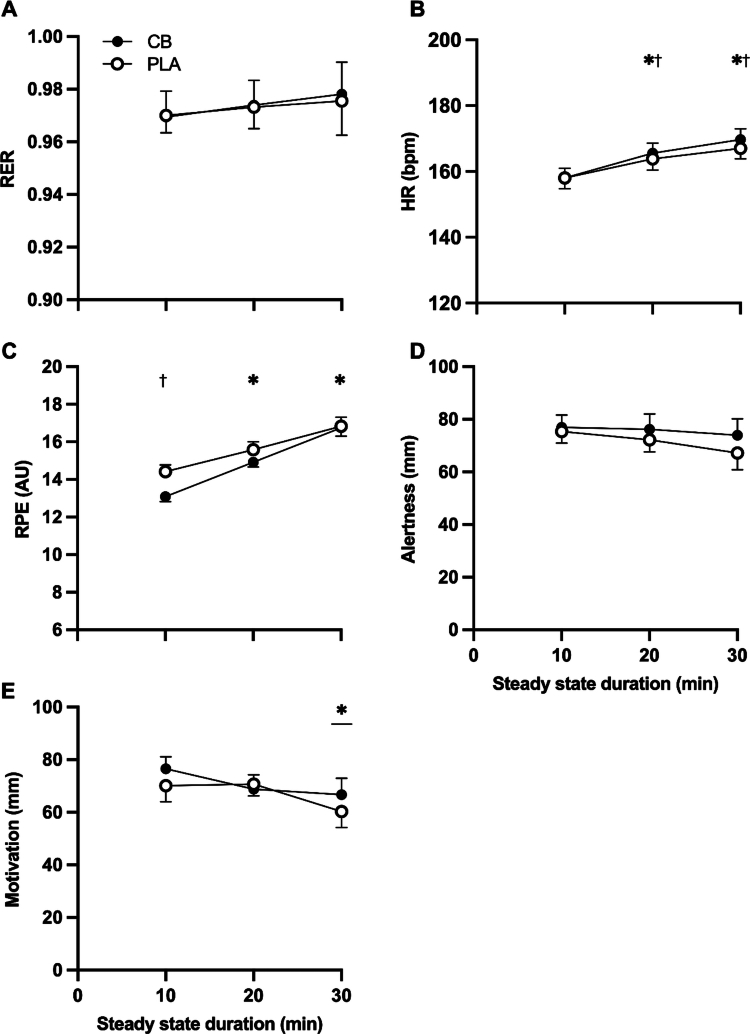
(A) respiratory exchange ratio (RER), (B) heart rate (HR), (C) rating of perceived exertion (RPE), (D) alertness, and (E) motivation during 30 min steady state exercise after consuming a multi-ingredient supplement containing 200 mg caffeine (CB; filled symbols) or placebo (PLA; open symbols) in trained cyclists (*n* = 12). Data analyzed by two-way ANOVAs. Significant time effect observed for motivation (*p* < 0.001; post hoc difference denoted by * *p* < 0.01 significantly different to 10 and 20 min). Post hoc differences within interaction effects denoted by ^†^ *p* < 0.05 CB significantly different to PLA, and *p < 0.01 significantly different to previous timepoint.

The average power output during the SS was 249 ± 12 W, which equated to 79% ± 1%V̇O_2max_. Relative V̇O_2max_ during SS was unaffected by CB (*p* > 0.05). There was no effect of trial order on %V̇O_2max_ attained during SS. Respiratory exchange ratio (RER; Figure 4A) remained stable throughout the SS and was unaffected by CB (all comparisons *p* > 0.05). Heart rate (HR; Figure 4B) increased over time in both PLA and CB, but to a greater extent with CB compared with PLA (interaction *p* < 0.05) at 20 min (1 ± 1%; post hoc *p* < 0.05) and 30 min of SS (2% ± 0%; post hoc *p* < 0.001). Rating of perceived exertion (RPE; Figure 4C) increased over time in both PLA and CB (*p* < 0.001), but was lower with CB compared with PLA (both supplement and interaction *p* < 0.05), in particular at 10 min of SS (9% ± 2%; post hoc *p* < 0.001). Alertness (Figure 4D) remained unchanged during the SS, and was unaffected by CB compared with PLA (all comparisons *p* > 0.05). Motivation (Figure 4E) was unaffected by CB (supplement and interaction *p* > 0.05), but decreased by 14% ± 4% from 10 to 30 min SS irrespective of supplement. Gastrointestinal discomfort remained unchanged during the SS, and was unaffected by CB compared with PLA (all comparisons *p* > 0.05).

### 3.4. Plasma metabolites

**Figure 5.**
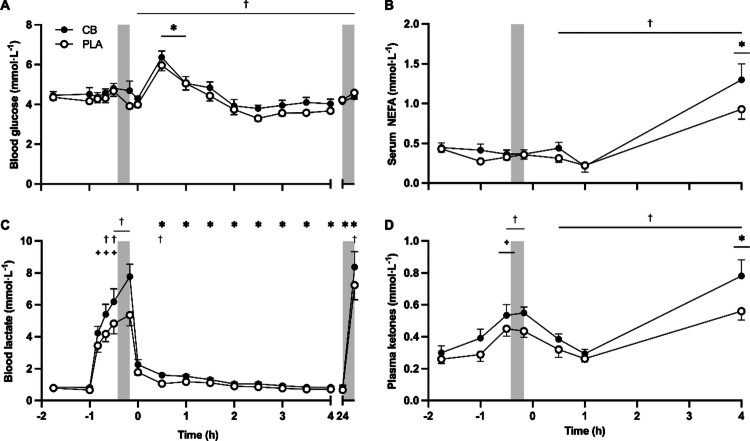
Concentrations of (A) blood glucose, (B) serum non-esterified fatty acids (NEFA), (C) blood lactate, and (D) plasma ketones, before and after consuming a multi-ingredient supplement containing 200 mg caffeine (CB; filled symbols) or placebo (PLA; open symbols) in trained cyclists (*n* = 12). Data analyzed by two-way ANOVAs, run separately for baseline to end of steady state, end of steady state to end of time trial, and recovery period, but plotted together for presentation purposes. During steady state: supplement by time interaction effect for blood lactate only denoted by ^+^ *p* < 0.001 significantly different to baseline during steady state within each group, ^†^ *p* < 0.05 CB significantly different to PLA at given timepoint. Main effect of time for plasma ketones presented, with post hoc differences denoted by * *p* < 0.01 end of SS significantly different to baseline. During time trial: main effect of supplement for blood lactate and plasma ketones, denoted by † *p* < 0.05 CB significantly different to PLA. During recovery: supplement by time interaction effect for blood lactate only, with post hoc differences denoted by *p < 0.05 significantly different to start of recovery during steady state within each group, ^†^ *p* < 0.05 CB significantly different to PLA at given timepoint. Main effect of supplement for blood glucose, serum NEFA and plasma ketones presented, denoted by † *p* < 0.05 CB significantly different to PLA. Main effect of time for blood glucose, serum NEFA, and plasma ketones presented, with post hoc differences denoted by * *p* < 0.05 significantly different to start of recovery.

Blood glucose concentrations did not differ between PLA and CB at baseline (Figure 5A). From baseline through SS and SS through TT, blood glucose concentration remained stable, and was unaffected by CB at any timepoint (all comparisons *p* > 0.05). During recovery and following ingestion of the carbohydrate beverage and second supplement, blood glucose concentrations increased by 51% ± 9% and 49% ± 9% with PLA and CB, respectively, at 30 min of recovery, and remained elevated at 60 min, before returning to concentrations observed at the start of recovery (time *p* < 0.001). Throughout recovery, mean blood glucose concentrations were greater with CB compared with PLA (4.44 ± 0.13 vs. 4.19 ± 0.10 mmol·L^−1^, respectively; supplement *p* < 0.05).

Serum NEFA concentrations did not differ between PLA and CB at baseline (Figure 5B). Compared with baseline, serum NEFA concentrations decreased at 0 and 30 mins of SS in both PLA and CB (time *p* < 0.05). Serum NEFA concentrations were unchanged throughout TT, and were unaffected by CB (all comparisons *p* > 0.05). Between 1 and 4 h of recovery, serum NEFA concentration increased from 0.22 ± 0.09 to 0.93 ± 0.13 mmol·L^−1^ and from 0.21 ± 0.04 to 1.30 ± 0.20 mmol·L^−1^ with PLA and CB, respectively (time *p* < 0.001). Throughout recovery (0–4 h post TT), mean serum NEFA concentrations were greater with CB compared with PLA (0.65 ± 0.08 vs. 0.49 ± 0.06 mmol·L^−1^, respectively; supplement *p* < 0.05).

Blood lactate concentrations did not differ between PLA and CB at baseline (Figure 5C). Compared with baseline, blood lactate concentrations remained stable until the onset of exercise (*p* > 0.05). Concentrations increased thereafter at 10, 20, and 30 mins SS, and to a greater extent with CB compared with PLA (interaction *p* < 0.01). At 20 and 30 min SS, blood lactate concentrations were 31% ± 7% and 29% ± 9% greater with CB than PLA, respectively (post hoc *p* < 0.05). Blood lactate concentrations exhibited a non-significant increase from 30 min SS to the end of the TT (time *p* > 0.05), remaining greater with CB than PLA (supplement *p* < 0.01). Upon the cessation of exercise, blood lactate levels decreased. This decrease from the end of the TT to 0 min of recovery was greater with CB than PLA (interaction *p* < 0.001). When compared to the onset of recovery, blood lactate concentrations continued to decrease over the subsequent 4 h recovery period. Prior to the second TT, blood lactate concentrations remained lower than at the onset of recovery (post hoc *p* < 0.001) from the previous day. Additionally, whilst pre-exercise lactate concentrations were unaffected by condition (PLA: 0.67 ± 0.06; and CB: 0.84 ± 0.13 mmol·L^−1^; post hoc *p* > 0.05), the second TT increased blood lactate in both conditions and to a greater extent with CB [7.23 ± 0.91 mmol·L^−1^ with PLA, CB 8.37 ± 0.97 mmol·L^−1^ (post hoc *p* < 0.001)].

Plasma ketone concentrations did not differ between PLA and CB at baseline (Figure 5D). At 30 min SS, concentrations increased from baseline (time *p* < 0.001) by 93% ± 26% and 107% ± 40% with PLA and CB, respectively. However, CB had no effect on plasma ketone concentrations during this timeframe compared to PLA (supplement and interaction *p* > 0.05). Before and after the first TT, plasma ketone concentrations were 21% ± 11% and 34% ± 12% greater, respectively, with CB than with PLA (supplement *p* < 0.05). Between 1 and 4 h of recovery, plasma ketone concentrations increased from 0.26 ± 0.02 to 0.56 ± 0.06 mmol·L^−1^ and from 0.29 ± 0.03 to 0.78 ± 0.10 mmol·L^−1^ with PLA and CB, respectively (time *p* < 0.001). Throughout recovery from 0 to 4 h, CB increased mean plasma ketone concentrations compared with PLA (0.49 ± 0.05 vs. 0.38 ± 0.03 mmol·L^−1^, respectively; supplement *p* < 0.05).

### 3.5. Muscle glycogen

**Figure 6.**
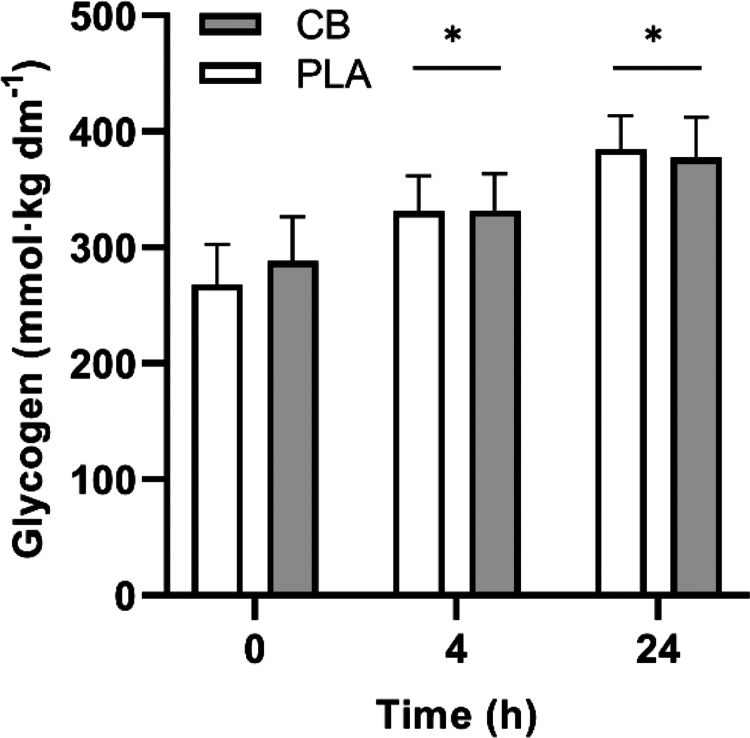
Muscle glycogen content of *m. vastus lateralis* at 0, 4, and 24 h of recovery after completing 30 min steady state cycling exercise followed by a 15 min time trial in trained cyclists (*n* = 8). Participants were given a multi-ingredient supplement containing 200 mg caffeine (CB; filled symbols) or placebo (PLA; open symbols) prior to exercise, and again at onset of recovery with 1 g·kg^−1^ carbohydrate. Data analyzed by two-way ANOVA. Main effect of time only, with post hoc differences denoted by * *p* < 0.001 significantly different to previous timepoint.

Muscle glycogen content (Figure 6) did not differ between PLA and CB at the onset of recovery (0 h). From 0 to 4 h, muscle glycogen content increased by 64 ± 13 and 44 ± 10 mmol·kg dry mass^−1^ with PLA and CB, respectively (time *p* < 0.001; post hoc *p* < 0.001). This increased further from 4 to 24 h of recovery, by 53 ± 8 and 45 ± 10 mmol·kg dry mass^−1^ with PLA and CB, respectively (post hoc *p* < 0.001). There was no effect of CB on muscle glycogen content overall (supplement *p* > 0.05) or at any timepoint (interaction *p* > 0.05).

### 3.6. Time trial performance

**Figure 7.**
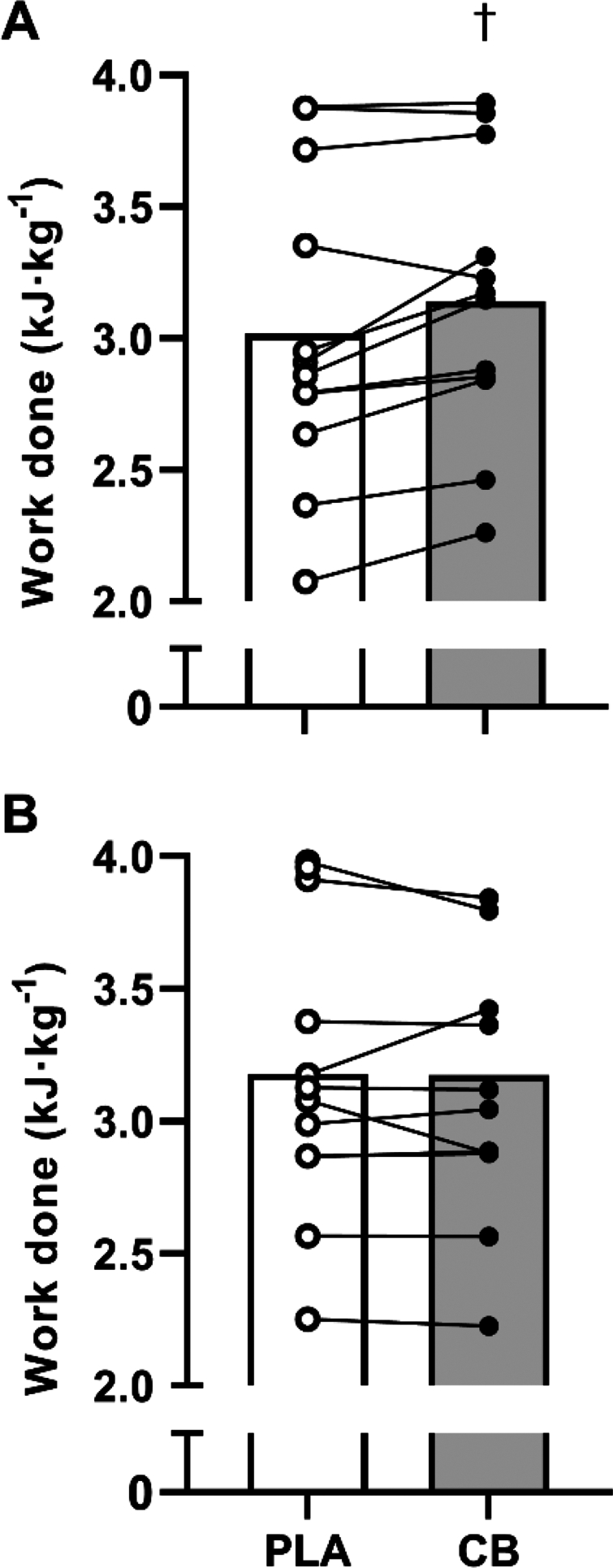
Work done during a 15 min time trial (A) after completing 30 min steady state cycling exercise and consuming a multi-ingredient supplement containing 200 mg caffeine (CB; filled symbols) or placebo (PLA; open symbols), and (B) after 24 h of recovery, in trained cyclists (*n* = 12). Data analyzed by paired samples *t*-test. Significant effect of supplement denoted by † *p* < 0.05 CB significantly different to PLA.

Work completed during the first TT, our pre-registered primary outcome, was greater with CB than PLA (3.14 ± 0.15 vs. 3.02 ± 0.17 kJ·kg^−1^; *p* < 0.05; Hedge's *g* = 0.8 [0.2,1.4]; Figure 7A). This equated to a mean power output of 3.49 ± 0.17 vs. 3.35 ± 0.19 W·kg^−1^, for CB vs. PLA, respectively (*p* < 0.05), and 4.6% ± 1.5% average individual improvement when supplemented with CB. After 24 h of recovery, work completed during the second TT was unaffected by CB (3.17 ± 0.16 vs. 3.18 ± 0.16 kJ·kg^−1^; CB vs. PLA, respectively; *p* > 0.05; Figure 7B). Accordingly, mean power output was unaffected by CB (3.53 ± 0.18 vs. 3.53 ± 0.18 W·kg^−1^; *p* > 0.05). There were no effects of trial order on TT performance (*p* > 0.05).

## 4. Discussion

Here, we hypothesized that ingesting a whole *Coffea arabica* coffee cherry extract containing low-dose caffeine and polyphenols before endurance exercise would reduce effort perception and improve performance. We further hypothesized that a second dose co-ingested with a carbohydrate beverage would increase muscle glycogen resynthesis post-exercise. Under these experimental conditions, the supplement, which contained 200 mg caffeine and approximately 15 mg of polyphenols, markedly raised plasma caffeine, paraxanthine, and chlorogenic acid concentrations and reduced effort perception during steady-state cycle exercise and increased total work performed during a time trial by ~5%. However, whilst plasma caffeine, paraxanthine, and polyphenol concentrations remained elevated throughout recovery, muscle glycogen resynthesis and performance in a subsequent time trial the next day was unaffected.

There are multiple reports that low doses of caffeine (~3 mg⋅kg bm^−1^), in isolation or formulated with other materials, may improve performance across a range of sporting activities involving bouts of sustained exercise [1]. Moreover, caffeine from brewed coffee, which may also contain certain polyphenols dependent on the degradation resulting from the roasting and brewing processes, has been reported to provide exercise performance benefits [4,5]. The whole coffee cherry from which the bean is derived is also rich in polyphenols. There is, however, a paucity of data addressing the ergogenic and potentially unique metabolic effects resulting from ingestion of low-dose caffeine from this non-roasted whole coffee cherry extract. Here, we report that a whole coffee cherry extract containing 200 mg caffeine (equating to 2.7 mg⋅kg bm^−1^ in our participants) and ~15 mg coffee cherry polyphenols increased plasma caffeine concentration markedly and improved time trial performance by ~5%. Given that previous meta-analyses report ~3% improvement in mean power output with doses of 3–6 [8] and 4–6 [20], but not 1–3 [20] mg⋅kg bm^−1^ of caffeine, it appears the coffee cherry extract used herein conferred (at least) a comparable increase to mean power output during the first time trial at 2.7 mg⋅kg bm^−1^, relative to what might be expected with higher doses previously reported in the literature. Indeed, a previous study using a similar experimental design with a significantly larger dose of a multiple-active-ingredient supplement, of which 200 mg of caffeine was one, also reported a similar degree of cycling performance improvement [14]. In both studies, the participants' rating of perceived exertion (RPE) was lower during prior steady-state exercise, suggesting that a mechanism of action was in part via a central effect. Indeed, caffeine and its major metabolite paraxanthine are potent adenosine-receptor antagonists [21], and data from rodent studies indicate that paraxanthine may offer additional ergogenic effects through augmenting dopamine release in the brain [22]. Given that exercise was performed in a fasted state, these findings may be particularly relevant to improving exercise capacity in situations where carbohydrate availability is low, including improving training quality during periods of carbohydrate periodization intended to improve metabolic flexibility [23].

Given that we also observed a greater plasma lactate and ketone concentration during steady-state exercise with coffee cherry extract supplementation in the present study, we cannot rule out a peripheral effect of the supplement as well. However, while it is well known that higher doses of caffeine increase circulating fatty acids, glucose, and lactate, the mechanism of action of the increase and any effect on exercise performance remains unresolved [24]. Moreover, performance in the second time trial was unaffected by prior consumption of the supplement (Figure 7B), despite elevated plasma caffeine, paraxanthine and lactate. This would fit with the suggestion that caffeine supplementation has a narrow window of action, improving performance when consumed >60 [3,14], but not >180 min [25,26] before a time trial, and that the increase in blood lactate is due to reduced clearance in non-exercising tissue, rather than altered metabolism in exercising skeletal muscle [24]. Despite significantly greater plasma lactate in the CB group following the TT, both groups progressed toward baseline levels within a similar timeframe during recovery. Our observation that plasma glucose was unaltered between conditions throughout rest and exercise diverges from other effects reported in the literature focused on pure caffeine supplementation. Several studies [27,28] have reported increases in plasma glucose during the 60-min rest period between ingestion and the onset of exercise, with additional increases during exercise. There have been further reports of increased blood glucose following doses of 3, 6 and 9 mg·kg^−1^ during >50 min of exercise to exhaustion at 85% V̇O_2max_ [7]. In our current study, however, plasma glucose remained unchanged by CB throughout exercise and was only observed to increase after ingestion of the carbohydrate beverage (~75 g dextrose) and a second CB dose containing 2.7 mg·kg^−1^ of caffeine. This increase was similar to that experienced by placebo. Subsequent differences between groups during recovery also indicated that CB resulted in higher average blood glucose during this period than PLA. This relative elevation served to blunt the magnitude of a potential hypoglycemic event (<3.9/4.0 mmol·L^−1^) as experienced by PLA. Further work is necessary to establish if these peripheral responses are a result of the complexation of coffee fruit polyphenols with caffeine. Nevertheless, the low dose efficacy and potential for improved metabolic stability could be of interest to athletes who are sensitive to more typical caffeine ingredients or, who are mindful of total daily caffeine exposure with carbohydrate consumption during long training sessions, tournaments or competitions.

Muscle glycogen is the predominant fuel source during high-intensity endurance exercise, providing ~60% of energy for exercise at ~80% V̇O_2max_ [29,30]. Indeed, throughout the steady-state exercise, mean RER in both trials was 0.97 (Figure 4A), indicating heavy reliance on carbohydrate oxidation for energy. Moreover, muscle glycogen content was ~268 and ~290 mmol·kg dm^−1^ following the first time-trial in PLA and CB (Figure 6), which is consistent with that typically reported after similar exercise protocols in this population [31], as well as previous reports that caffeine does not influence muscle glycogen utilization during exercise [24]. Our secondary hypothesis that a coffee cherry extract would promote glycogen resynthesis was based on prior reports that larger doses of 8 mg⋅kg bm^−1^ caffeine can influence substrate handling both at rest [32] and during exercise [33,34], and that lower doses of caffeine, when uniquely complexed with polyphenols [11], may have unexpected metabolic effects. However, here we report that when taken twice, both before and after exercise, providing a total of 400 mg caffeine (or 5.4 mg·kg bm^−1^ in our cohort), this supplement did not improve muscle glycogen resynthesis when measured 4 h following consumption of 1 g·kg bm^−1^ carbohydrate, or after 24 h with an additional mixed macronutrient meal (containing 1.2 g·kg bm^−1^ carbohydrate), which is in line with some [35–38], but not all [9,10,39], previous work. Explanations for this discordance are lacking, especially as caffeine appeared to support muscle glycogen resynthesis when total carbohydrate provision was lower and/or provided over a shorter time, i.e. 4 g·kg bm^−1^ over 4 h [9,10] versus 5–7 g·kg bm^−1^ over 5–6 h [35,36]. We also acknowledge the possibility that habitual dietary intake over a prolonged period (beyond the day prior to, and of, the study visits) may influence subsequent carbohydrate metabolism [40], though we are unable to determine the influence of this in the present study as we did not control for, or capture longer-term habitual intakes. However, beneficial effects of caffeine supplementation on glycogen resynthesis are usually observed following exercise to exhaustion with significant glycogen depletion to ≤100 mmol·kg dw^−1^, which is known to accelerate muscle glycogen resynthesis [41]. Indeed, as a result of the study design implemented here, muscle glycogen was only reduced to around 280 mmol·kg dw^−1^ following exercise in the present study, and resynthesized by only 50 mmol·kg dw^−1^ over 4 h across both groups, rendering it likely that any beneficial effect of caffeine and/or polyphenols would have been missed and requires further research.

Caffeine and chlorogenic acids are major constituents of the whole coffee cherry fruit (fruit and bean). This particular whole *Coffea arabica* cherry extract has previously been demonstrated to possess novel complexation properties that are not attainable by formulating mixtures of highly purified extracts of caffeine and chlorogenic acids alone [11]. Chlorogenic acids found in coffee cherries include caffeoylquinic, feruloylquinic, p-coumaroylquinic, dicaffeoylquinic, diferuloylquinic, di-p-coumaroylquinic and feruloylcaffeoylquinic acids [6]. Here, we report for the first time that several of these acids, particularly 4-caffeoylquinic acid, can appear in the circulation and peak around 1.5 h after whole coffee cherry extract consumption, which fits with the plasma profile and concentration of other chlorogenic acids and similar compounds that have been associated with beneficial effects on metabolism and vascular function after blueberry extract ingestion [42]. Indeed, such studies often report a second peak in circulating polyphenol metabolites some 6 h after fruit extract ingestion, which also appeared to occur in the present study. Whether the increase in chlorogenic acids following coffee cherry ingestion in the present study affected metabolism or performance cannot be determined with certainty based on these data. However, while changes in circulating substrates are routinely observed following doses of caffeine greater than 3 mg·kg bm^−1^ [24], we are not aware of any reports of greater circulating lactate and ketones during exercise, or greater circulating NEFA and ketones after 4 h of recovery, following low-dose caffeine ingestion alone. These alterations to substrate circulation occurred without changes to circulating glucose during rest and exercise while also maintaining more stable circulating glucose during the recovery period. Whether these outcomes reflect a metabolic effect of caffeine complexed with coffee cherry chlorogenic acids requires further investigation.

In summary, we report that ingestion of a whole *Coffea arabica* cherry extract containing 200 mg caffeine and 15 mg of polyphenols reduces effort perception during steady-state exercise and increases total work performed during a subsequent ecologically valid time trial. However, under these experimental conditions, such formulations do not appear to enhance muscle glycogen resynthesis and recovery when muscle glycogen has not been significantly depleted, and moderate carbohydrate is consumed post-exercise. Further work is required to explore whether other micronutrients in this extract from whole *Coffea arabica* coffee cherries can affect exercise metabolism, performance, and recovery.

## Data Availability

Original data arising from this research are available directly from Professor Francis Stephens upon request.
